# Software Architecture and Framework to Develop NFC-Based Applications

**DOI:** 10.3390/s18082654

**Published:** 2018-08-13

**Authors:** Ricardo Tesoriero, Jose A. Gallud

**Affiliations:** Faculty of Computer Science Engineering, Computing Systems Department, University of Castilla-La Mancha, 02071 Albacete, Spain; ricardo.tesoriero@uclm.es

**Keywords:** near field communication, model-driven development, mobile computing

## Abstract

Applications are employing Near Field Communication (NFC) technology to interact with physical objects by using NFC tags. The architecture to implement these kinds of applications varies according to non-functional requirements such as the physical environment where the application is running, the flexibility to adapt the information to be manipulated through physical objects, etc. To cope with these challenges, this paper proposes a Model-driven Architecture (MDA) where designers are able to model the configuration of the system according to NFC based system requirements. Through a model-to-text transformation process, the MDA also provides developers with templates of source code for the applications that support the system. The proposed MDA process defines a Platform Independent Model (PIM) which supports a Domain Specific Language (DSL) implemented as an Eclipse platform plugin that provides designers with a graphical model editor, and a model to text transformation, to generate the source code templates using the Acceleo transformation language. The paper also presents the Albacete Photo Gallery case of study to illustrate the modelling process. The main benefit of the proposal is that it allows developers to create mixed environments where the availability and flexibility of NFC based interaction systems can be easily configured, extended and maintained.

## 1. Introduction

The use of tags to interact with physical objects using electronic devices is widely deployed, and the technology employed to develop these systems has been evolving during the last few decades.

One of the most popular methods to interact with objects is based on numeric codes. To interact with physical objects, users introduce codes that are associated with physical objects in a keyboard/keypad, or they just identify the code on a touch screen. This way of interaction is very common in auto-service supermarkets to purchase fruits or free-flowing products. It is also a very popular method to access information in museums or art expositions where tourists introduce codes associated to rooms or art objects into electronic devices to hear the explanation related to them.

The lack of reliability of codes that are manually introduced by users leads to the introduction of barcodes or QR Codes [1]. Both of these methods avoid the need for users to introduce codes, or small pieces of information such as URLs manually. However, from the interaction perspective, this technology “pollutes” the physical object with information that is exposed to the user, but is not intelligible. This matter becomes worse when the physical object exposes information that is relevant to the user, and it is scattered with information that is only readable by electronic devices. For instance, due to physical space restrictions, subway maps expose station names in one, or at most two languages. It is almost impossible to attach any extra information for codes that are read by electronic devices. The same situation occurs while touring across museums or art galleries where explanation panels are written in one or two languages.

Near Field Communication (NFC) technology allows devices to read information that is not visible to users. Therefore, this technology is the most suitable to replace barcodes and QR Codes because it does not “pollute” physical objects with information that is only readable by machines.

This technology has been employed in different domains. An example of the use of this technology in the tourism domain is presented in [2]. Another example where this technology is used to encourage the promotion of the environmental sustainability is presented in [3]. Finally, the adoption of this technology in the learning domain is presented in [4].

The goal of this proposal is the definition of a Model-driven Application (MDA) [5] capable of configuring an NFC-based system according to non-functional requirements such as availability, flexibility and reliability and generating templates of source code that support a system’s applications.

This paper is structured as follows: Section 2 presents the related work. Section 3 presents the characteristics of NFC based applications, Section 4 presents the MDA that supports the development of NFC based applications, Section 5 presents the framework that is employed to develop NFC based systems, Section 6 presents the Albacete Photo Gallery Application case of study and Section 7 presents the benefits of the proposal. Finally, in Section 8, the conclusions and future works are exposed.

## 2. Related Work

The Model-driven Architecture (MDAs) [5] approach proposed by the Object Management Group (OMG) [6] in 2011 presents a set of tools to abstract these common elements to improve the software development. This solution gives a leading role to models in the software development during all phases (i.e., inception, design, building, development, and maintenance).

The main reason behind this approach is the constant evolution of the software technologies. Following a traditional development approach, the functionality code and the implementation technology code are interweaved. Consequently, when the technology is enhanced, the functionality is rewritten using the new technology.

Web applications are a good example of this scenario since heavyweight server applications are being gradually upgraded to lightweight applications using REpresentational State Transfer Application Programming Interfaces (ReST APIs) [7] Web services implementing.

Under these scenarios, MDAs introduce abstraction levels to promote the software reuse by emphasizing the design-time interoperability [8].

This kind of interoperability is possible due to the specification of Platform Independent Models (PIMs) that enable developers to separate the specification of the application functionality from the technology that implements it.

Thus, it is possible to reuse the specification of the application functionality for different implementation technologies. Moreover, this functionality can be executed to different hardware and software platforms only with minor changes.

The source code of applications is automatically derived from models using model transformations [5].

In summary, the use of the MDA technology enables the generation of multi-platform applications from PIMs.

The core of the MDA infrastructure is defined in terms of the following OMG standards: Unified Modeling Language (UML) [9], the Meta Object Facility (MOF) [10], XML Metadata Interchange (XMI) [11] and the Common Warehouse Metamodel (CWM) [12], which were successfully used in the modelling and development of modern systems.

From the Human–Computer Interaction perspective, we can find different approaches that make use of models to generate user interfaces.

An early approach that employs models to generate user interfaces is presented in [13].

In recent years, other approaches such as [14,15,16] have also encouraged the use of models to develop multi-modal user interfaces.

However, none of them formalizes the definition of NFC-based applications using OMG compliant metamodels. Nevertheless, there are several works that use Model Driven Development (MDD) techniques based on MDAs to develop Web applications [17,18,19].

## 3. Interacting with Physical Objects Using NFC Technology

The NFC technology allows devices to read information that is stored in NFC tags providing users with the ability to retrieve information just by approaching the mobile device reader close to the NFC tag that contains the information (2 cm to 5 cm).

NFC tags store an identification code that allows application to identify the NFC tag. Depending on the tag product, the tag identification can be duplicated. For instance, ISO 14443 [20] Type A tags with 4-byte serial numbers allows duplications because there is no clear scheme to divide the available range of serial numbers among the various manufacturers. However, ISO 14443 Type A tags with 7-byte serial number and ISO 15693 [21] tags allocate a block of serial numbers for each manufacturer. Within that block, manufacturers guarantee that they use each serial number only once.

In addition, there are some NFC tags that are capable of storing data, which can be read and written by devices. This information can also be encrypted in order to provide extra security to the data stored in the tags. Therefore, one way to avoid tag duplication is using the tag memory to store a code that is unique for the application.

To interact with physical objects, users approach the mobile device NFC reader next to the “area of interest” or “hot spot” of the physical object. As a result, the application reacts to the user gesture.

**Figure 1:**
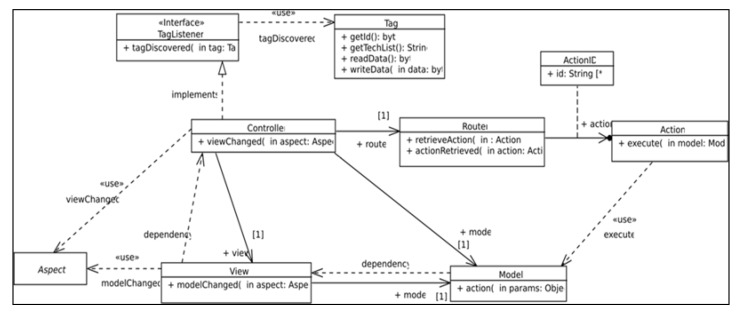
UML class diagram that depicts the software infrastructure required to support NFC applications.

The software infrastructure to support this kind of interaction is defined in the class diagram depicted in Figure 1. This diagram shows the realization of the Model View Controller (MVC) software architecture pattern where the *Controller* realizes the *TagListener* interface that defines the *tagDiscovered(tag)* operation, which is executed when the mobile device NFC reader discovers an NFC tag. The *Controller* delegates to the *Router* the responsibility for finding the action (represented by an instance of the *Action* class) to be performed on the model. This action plays the role of *Command* in a realization of the *Command* design pattern [22] where the receiver role is played by an instance of the *Model* class. As result of this execution, the model notifies its changes to its dependents by executing the *modelChanged(aspect)* operation. As *View* class instances depend on *Model* class instances, views receive notifications, which are forwarded to view dependents.

**Figure 2:**
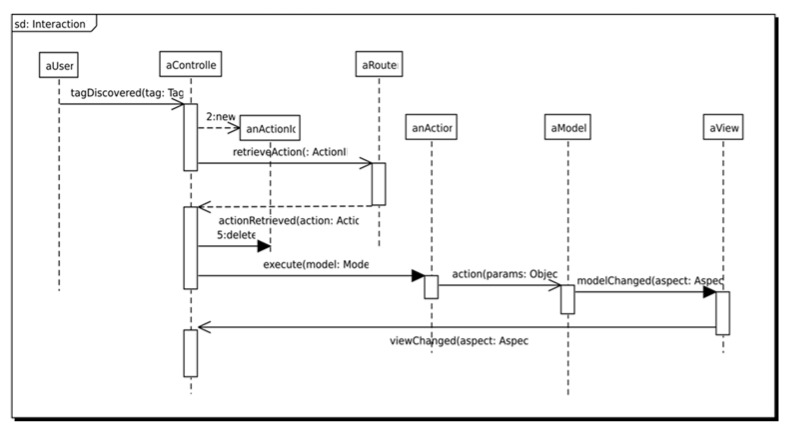
UML sequence diagram that depicts the sequence of interactions among software architecture components when an NFC tag is discovered.

Figure 2 defines the sequence of operations that is executed by the system when an NFC tag is discovered by the NFC reader of the mobile device. The diagram shows four asynchronous operations: *retrieveAction*, *actionRetrieved*, *action* and *modelChanged*. The first two operations represent the possibility of configuring the *Router* as local or remote. While a local router allows offline applications to define fixed associations between physical objects and actions; remote routers allow the change of the association of physical objects to actions dynamically. The last two operations represent the possibility of defining a local or a remote model. While local models are used to develop offline applications; remote models allow the definition of dynamic application behaviour using a centralized server.

## 4. The Model-Driven Architecture

This paper proposes an MDA to develop NFC based applications. This architecture defines three points of view. The first one defines a Platform Independent Model (PIM), and the second one defines a Platform Specific Model (PSM). In addition, the third one represents the Implementation Specific Model (ISM) or the source code.

The MDA implementation follows the OMG [6] standard, which allows the re-use of models while ensuring the cross-platform interoperability and platform independence. For instance, the OMG defines the XMI (XML Modelling Interchange) format [11] to store models allowing developers to re-use models among different tools that support this technology.

The technology employed to support these standards is based on the Eclipse platform, which provides frameworks that support OMG standards. For instance, the Eclipse Modeling Framework [23] to support model creation and manipulation, the Eclipse Modelling Project [24] to support the creation of graphical model editors, the Eclipse OCL Project to support the Object Constraint Language (OCL) [25] and the Acceleo [26] standard to support the model to text transformation.

### 4.1. The Platform Independent Model

**Figure 3:**
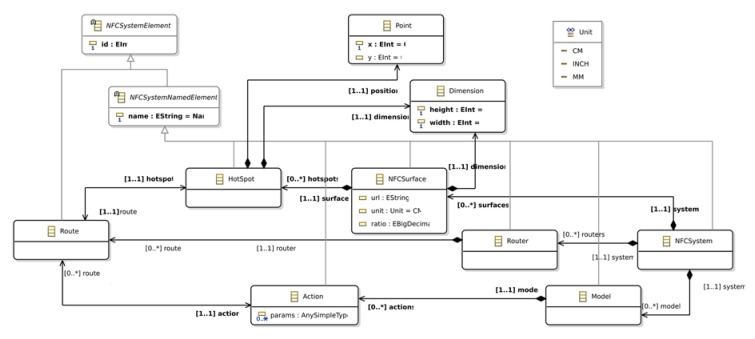
The Platform Independent Model point of view metamodel.

The Platform Independent Model (PIM) defines the abstract syntax of the Domain Specific Language (DSL) that describes the NFC based application to be generated. Figure 3 depicts the metamodel employed to define NFC based applications.

The PIM defines the *NFCSystem* as the “root” metaclass that represents the whole NFC system. The system is composed by a set of *NFCSurface* instances that represent the surface of physical objects. It also contains a set of *Model* instances that represent the application behaviour. Finally, it contains a set of *Router* instances that represent the associations between *NFCSurface* instances and *HotSpot* instances, and the associations between *Model* instances and *Action* instances.

*NFCSurface* instances represent the relationship between physical objects and their virtual representation. The virtual representation of the physical object is an image that is stored as a URL in the URL property. To define the size relationship between the virtual representation and the physical object, the *NFCSurface* instance defines a *Dimension* instance that specifies the height and width in pixels of the virtual representation of the object. To transform this virtual dimension to a physical magnitude, we employ the ratio and unit properties. The ratio defines how many pixels of the virtual representation represent a physical unit. For instance, 100 px in an image represent 0.5 m if the ratio property is set to 2 and the unit is set to CM (centimeters).

*HotSpot* instances represent regions or areas of the *NFCSurface* that are tagged. In order to identify these areas, HotSport instances define a position and a dimension in pixels. The correspondence between the physical area and, the virtual dimension and position, is defined by the unit and ratio properties that are defined in the *NFCSurface* that contains them.

The metamodel supports the definition of different *Model* instances to represent the application behaviour. It allows designers to distribute the behaviour of the system easily. Each model defines a set of *Action* instances that can be performed on it.

HotSpot instances are linked to *Action* instances by the means of *Route* instances. A *Route* represents a link between a physical surface and a computing action. *Route* instances are grouped in *Router* instances that define the how *Action* and Hot *Spot* instances are linked.

### 4.2. The Platform Specific Model

The Platform Specific Model (PSM) is defined using the marking model. This way of defining the metamodel has two advantages: it allows designers to re-use the marking model for different PIM; and it allows designers to re-use the PIM with different marks.

**Figure 4:**
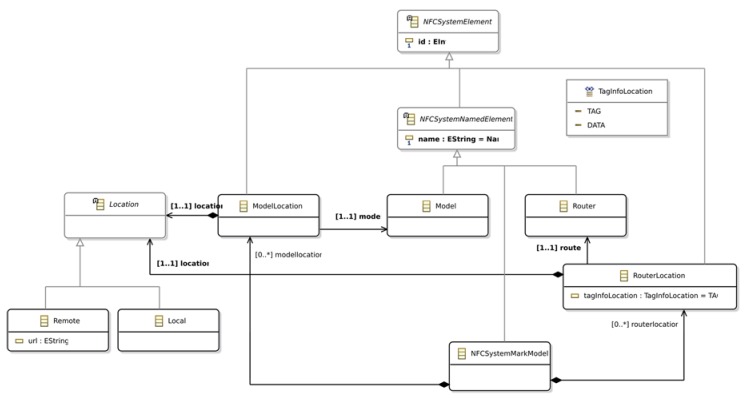
Marking model to define the Platform Specific Model.

The marking model used to define marks is depicted in Figure 4. It defines the *ModelLocation* and the *RouterLocation* metaclasses. Both metaclasses define a *Location*, which is either *Local* or *Remote*. If the *Location* is *Remote*, then the URL that addresses the *Model* or *Router* should be defined. In addition, the *RouterLocation* defines the *tagInfoLocation* property that is used to determine if the routing to the action is performed using the tag *id* or the tag *data*.

Consequently, this metamodel allows the definition of four different types of *Router* configurations and two different types of *Model* configurations. The advantages of defining different platform configurations allow designers to specify which parts of the system are available online and which parts are available offline improving the reliability of the system. In addition, it allows designers to decide which parts of the system should be kept on the server side due to security reasons. Finally, it also allows designers to define which parts of the system must be stored on servers because they are regularly modified, and they should be kept on the client to reduce the network traffic.

## 5. The NFC-Based System Framework

Our proposal presents a framework that supports the NFC system by means of three different applications.

### 5.1. User Applications

The goal of User Applications (UApps) is providing final users with a tool to interact with physical objects using an NFC based application.

These applications are responsible for reading NFC tags, using the *Router* to find out the *Action* to be performed on the *Model*, executing the *Action* on the *Model* and updating the User Interface (UI) according to changes performed on the model.

If the configuration is set to define an offline system (*Router* and *Model* location properties are set to *Local*), UApps are in charge of implementing the *Router*, the *Model* behaviour and containing the whole application resources. Otherwise, UApps delegate this responsibility to *Server Applications* by means of *Server Proxies* depending on the value of the *model* and *router* location properties.

From an implementation perspective, this proposal employed the Android technology as a platform to generate the source code of the UApp. The implementation defines an Android Activity that is in charge of forwarding the discovery of NFC tags to a WebView component. The WebView sends the tag information to the Controller, which is coded in JavaScript and updates the view, which is coded in HTML and CSS.

### 5.2. Server Applications

The Server Applications (SApps) are required to provide client resources (i.e., audio, video, text, images, etc.) and to implement remote *Models* and *Routers*. SApps implement *Routers* as services where UApps queries the service sending the NFC tag *id* or NFC tag *data* as a parameter to retrieve the action to be performed on the *Model*.

This proposal implements the *Router* service using a ReST API that allows UApps to get the action associated with a tag *id* or *data* using the *path/routername/id/:id or/routername/data/:data* where the *routername* is the name of the router service, and *id* and *data* contain the information of the tags.

The implementation of the *Model* also employs a ReST API that allows UApps to execute actions on the model. The server path to execute actions on the server is */modelname/action* where *modelname* identifies the model on the server, and the *action* defines the action to be performed. The parameters are passed to the server using the body of the POST request that executes the action. The code generated to create the SApp is JavaScript and the technology employed to support server application is Node.JS and MongoDB.

### 5.3. The Configuration Application

The deployment of an NFC based system requires a configuration phase where NFC tags that are the link to the physical space are associated with a computational action. This link is performed by the Configuration Application (CApp). This link is performed in different ways according to the configuration of the Router.

When the value of the location property is *Remote* and the value of the *tagInfoLocation* property is set to *TAG*, the CApp associates the tag *id* to the action in the SApp. However, if the value of the location property is *Remote* and the value of the *tagInfoLocation* property is set to *DATA*, it associates the code on the tag *data* to the Action in the SApp.

When the location property is set to *Local* and the value of the *tagInfoLocation* property is set to *TAG*, the CApp associates model actions to tag *ids* in a JSON file that acts as a database that should be added to the UApp. However, if the location property is set to *Local* and the value of the *tagInfoLocation* property is set to *DATA*, the CApp writes the action on the tag. Therefore, the tag contains the action to be performed.

Both *Local Router* configurations have advantages and disadvantages. While writing the action on the tag allows developers to change the association between tags and actions without modifying the UApp, it proved to be less secure because it can be easily modified.

## 6. The Photo Gallery Case of Study

This section describes an example of the modeling and the development of the Albacete Photo Gallery Application (APGA) using the proposed architecture and framework.

### 6.1. The APGA Platform Independent Model

The PIM for the APGA was created by means of an Eclipse feature that was created using the EMF [23] and GMP [24] eclipse frameworks. The PIM was created using a combination of textual and graphical syntax that represents the concrete syntax of a DSL that follows the abstract syntax defined by the metamodel depicted in Figure 3.

**Figure 5:**
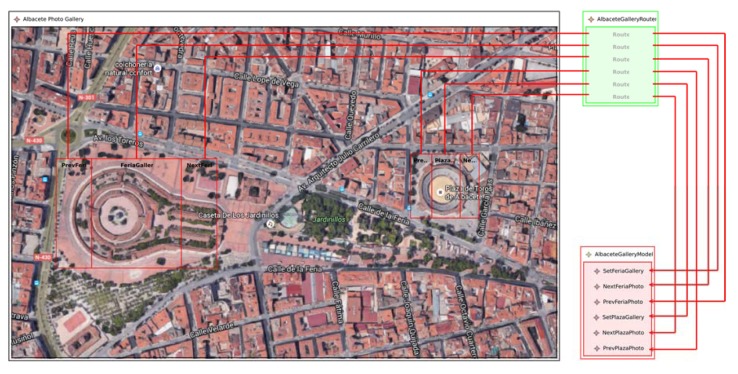
The platform independent model of the Albacete photo gallery application.

Figure 5 depicts the PIM of the APGA. The *AlbaceteGalleryModel* defines six *Actions* to manipulate two photo galleries (*FeriaGallery* and *PlazaGallery*). While the first three Actions allow users to go to the first (*SetFeriaGallery*), the next (*NextFeria*) and previous (*PrevFeria*) photos of the Feria Gallery; the last three Actions allow users to go to the first (*SetPlazaGallery*), next (*NextPlaza*) and previous (*PrevPlaza*) photos of the Plaza Gallery.

The Albacete Photo Gallery *NFCSurface* defines six *HotSpots* to manipulate both galleries. The interaction mechanism employed to browse the photo galleries simulates the typical Web photo gallery browser where users click on the right side of the photo to go to the next photo and click on the left to go to the previous one. To browse the gallery using an NFC-based application, users retrieve the next and previous photos in the gallery by approaching the reader over the right or left side of the building, respectively.

Therefore, three *HotSpots* were defined for each gallery. While the *PrevFeria*, the *FeriaGallery* and the *NextFeria* are defined to browse the Feria Gallery; the *PrevPlaza*, the *PlazaGallery* and the *NextGallery* are defined to browse the Plaza Gallery.

Finally, the *Router* assigns the *HotSpots* to *Model Actions* by the means of *Route* instances. Although this example shows a one-to-one correspondence between *HotSpots* and *Actions*, the model allows one-to-many relationships between *Actions* and *HotSpots*.

### 6.2. The APGA Platform Specific Model

The next step to generate the application source code is the definition of the APGA Platform Specific Model. In this case, we have not developed a graphical model editor; we have created a reflexive editor instead. The main reason lays on the simplicity of the metamodel (see Figure 4) and models that are created.

**Figure 6:**
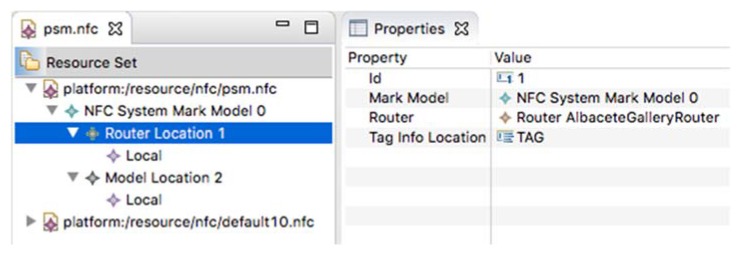
The Platform Specific Model of the Albacete photo gallery application.

For the sake of simplicity, the configuration of the system platform sets the *Router* as *Local* component, which means that it is part of the UApp. In addition, this configuration sets the *tagInfoLocation* in the tag data. Therefore, the *Action* to be performed on the *Model* is stored as data on the tag. Regarding the model, the *ModelLocation* is set to *Remote*; therefore, the *Model* is accessed via proxy from the UApp to the SApp. The PSM of the APGA is depicted in Figure 6.

### 6.3. The PSM-to-Text Transformation

The result of transforming the PSM to source code is the generation of the UApp (containing the Router and the Model proxy), the SApp (containing the Model) and the CApp, which is in charge of set Actions on the tag data.

The UApp employs four different programming languages to carry out its task. While Java and XML are employed to support the Android NFC infrastructure, HTML and JavaScript are employed to implement the model, view and controller of the application.

The code that connects the Android NFC infrastructure to the HTML and JavaScript is the same for all applications and it is defined by the API composed by the *tagDiscovered(tag)* and the *writeTag(tag)* functions.

**Figure 7:**
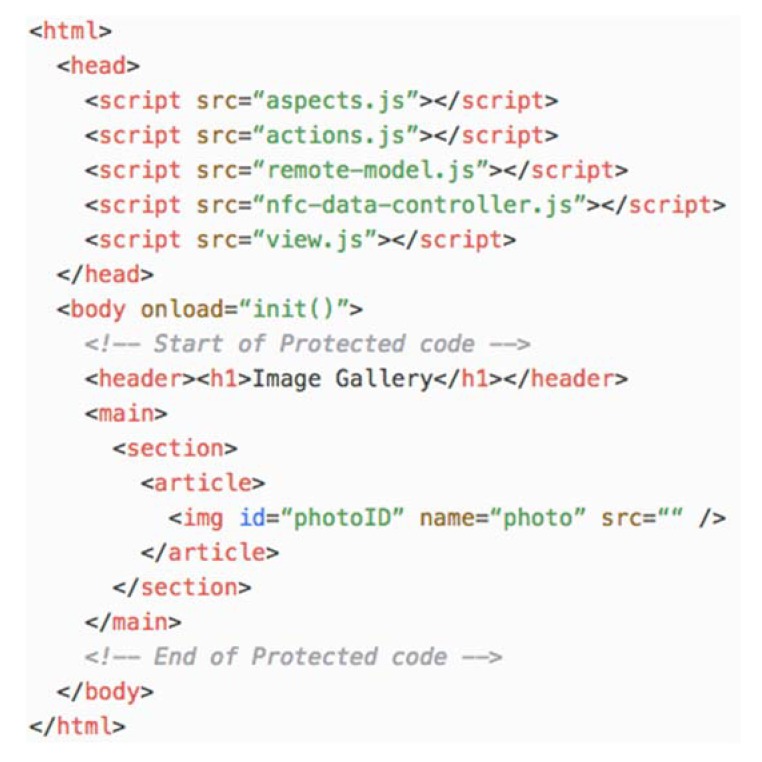
The HTML code for the view of the APGA UApp.

The HTML code generated for the view of the UApp is depicted in Figure 7. This code defines the links to the JavaScript code that implements the model, view and controller of the UApp. It also defines a protected section, which is delimited by the “Start of protected code” and “End of protected code” comments, where developers define the UApp user interface. This protected code is depicted in bold.

The behaviour of the view is defined in the view.js JavaScript file. It contains the view and controller objects that are initialized by the *init()* function when the HTML document body content is loaded.

The *init()* function creates the model proxy and binds the *modelChanged(info)* function to it. The *modelChanged(info)* function is called when the model changes. The info parameter provides information about the change including the aspect of the model that was affected (i.e., *Aspect.PHOTO_CHANGED*). All aspects are defined in the aspects.js file.

**Figure 8:**
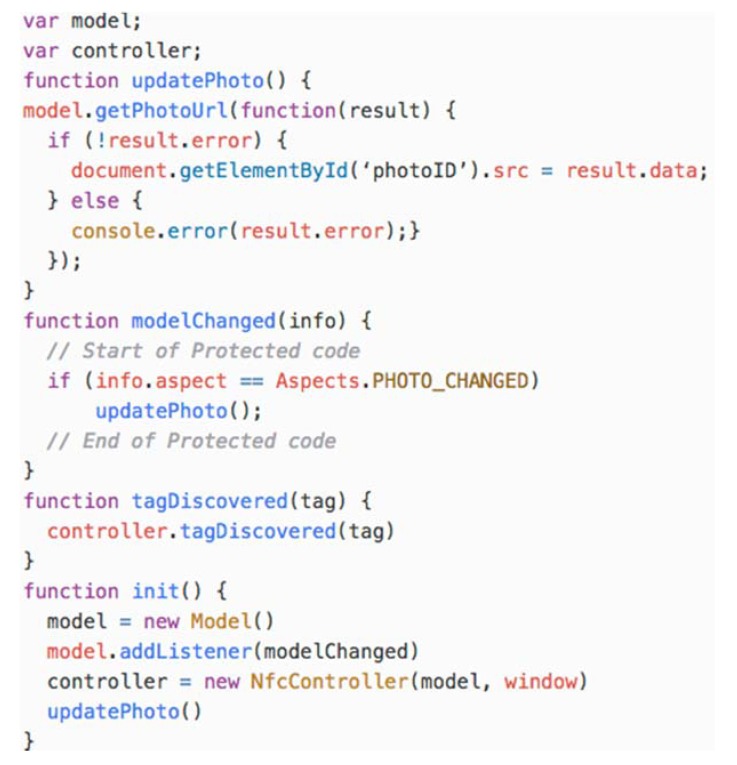
The view.js JavaScript file.

The view.js file also defines *the tagDiscovered(tag)* function that is called from the Android NFC infrastructure and it is forwarded to the controller. Figure 8 shows the view.js file. The code in bold is added by the developer to customize the behaviour of the view.

**Figure 9:**
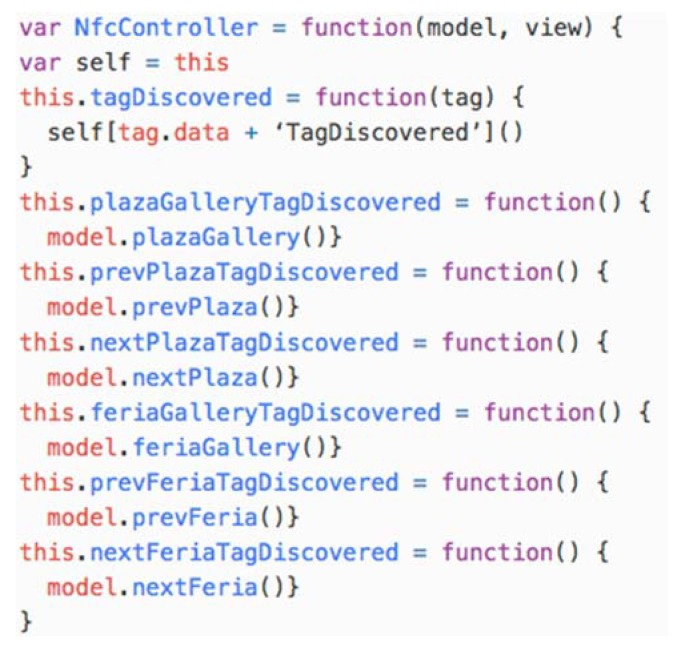
The nfc-data-controller.js JavaScript file.

The router is implemented as part of the application controller in the nfc-data-controller.js JavaScript file (see Figure 9). The *tagDiscovered(tag)* function is executed when tags are discovered by the Android NFC infrastructure, and, according to the PSM configuration, this operation finds the action to be performed and executes it on the model.

**Figure 10:**
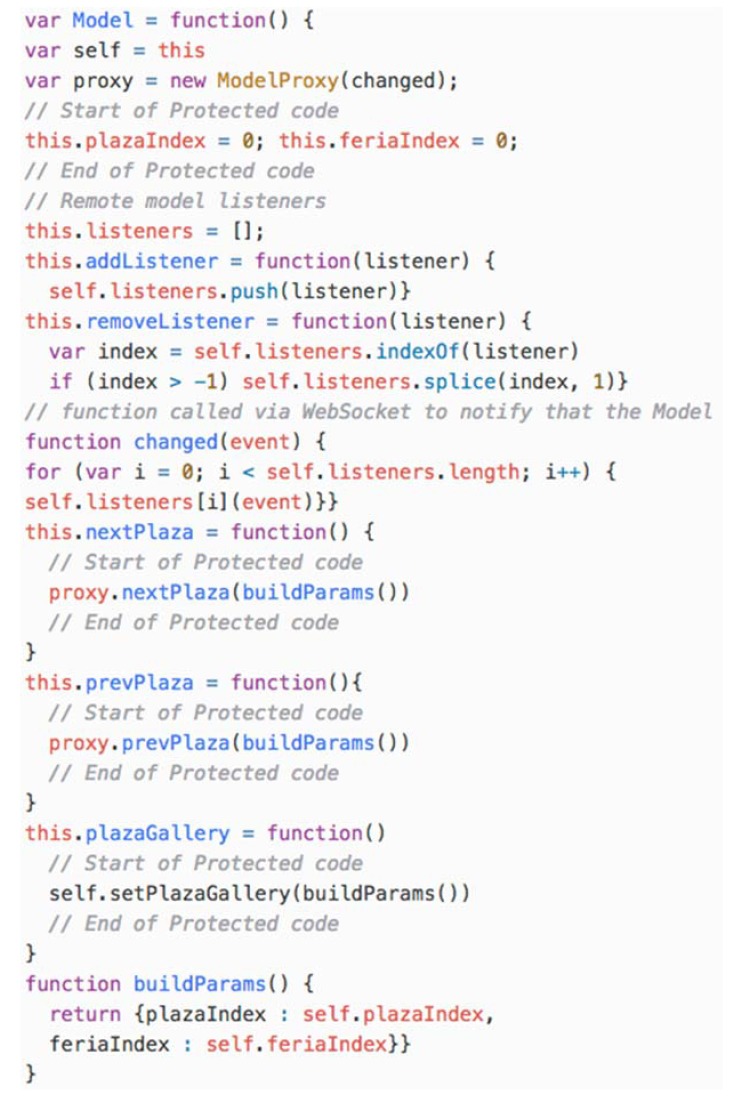
Fragment of code generated by the transformation for the remote model configuration.

The last software component that is part of the UApp is the *Model*. According to the model configuration, the Model is executed remotely; therefore, the UApp is connected to the Model via a Model Proxy. The most relevant fragment of code generated by transformation is depicted in Figure 10. The *Model* defines two local variables to store the local state of the model (*plazaIndex* and *feriaIndex*). In addition, it creates a *ModelProxy* that is in charge of connecting this component to the remote model server by calling the appropriate path address according to the action to be performed. The *ModelProxy* is also in charge of associating the *changed()* function to the remote model *WebSocket* that notifies remote model changes. Finally, it defines a set of functions that represent the execution of local model action behaviour that should be implemented by the developer.

**Figure 11:**
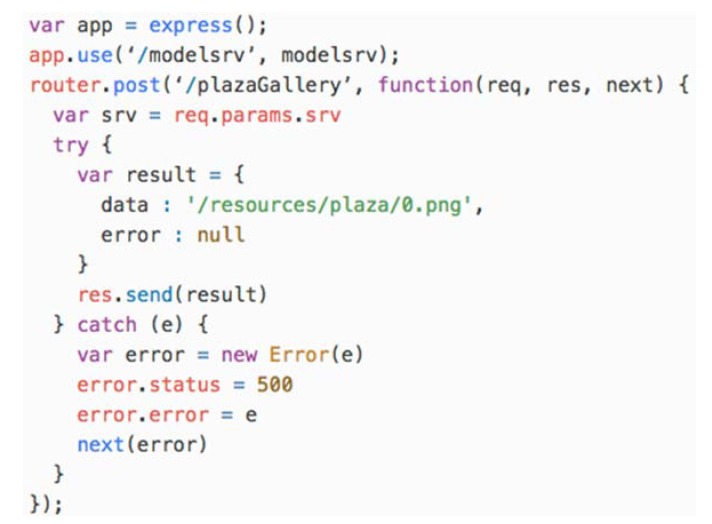
Fragment of code generated by the transformation for the remote model configuration.

The SApp is implemented in JavaScript using the Node.JS programming platform. The transformation generates a ReST API where each action is mapped to an address. A code example for the *plazaGallery Action* is depicted in Figure 11.

The CApp that is generated through the transformation process is very similar to the UApp because it employs the same Android NFC infrastructure and a WebView to interact with physical objects.

The main difference lays in the view and controller of the application. To assign tags to actions, the CApp defines an *ImageMap* on the HTML document that is supported on the WebView. The ImageMap HTML tag allows the definition of interactive regions on an image. Therefore, the application generates an *ImageMap* where the image represents the *NFCSurface* defined in the PIM, and the regions represent the *HotSpots* that were also defined in the PIM.

To assign NFC tags to *HotSpots*, the person in charge of the deployment has to discover the tag by approaching the reader to the tag and selecting the region on the image map that is assigned to the desired *HotSpot*.

## 7. Discussion

This section discusses the benefits and weaknesses of the proposal. In [27], it is explained that a good model has to cover the following five characteristics: (1) abstraction, (2) understandability, (3) accuracy, (4) predictiveness, and (5) inexpensiveness:

- *Abstraction*: A model of a system should reduce the details of the system it represents.
- *Understandability*: A model should be easier to understand than the system it models.
- *Accuracy*: While abstraction may summarize or hide important details, the meaning of these details should not be altered by a model.
- *Predictiveness*: A model should be executable in order to help developers predict specific system behavior.
- *Inexpensiveness*: A model must be inexpensive to produce.

### 7.1. Abstraction

The proposed MDA architecture allows developers to focus on the special features of the NFC-based application that they want to develop. 

The PIM defined by the MDA allows designers to configure how the physical surfaces are identified and where model actions are executed independently from the platform used to implement the system. Therefore, these models can be reused using different model-to-model transformations to generate different PSMs for different platforms.

These PSMs can be used to generate different ISMs using model-to-text transformations to generate systems source code in different programming languages (e.g., XML, CSS, HTML JavaScript, Java, Android, etc.)

The definition of these levels of abstraction reduces maintenance costs because changes and errors fixed on the PIM level are automatically propagated to all implementations.

The use of models also enables the model validation to check domain model at design time reducing testing effort.

### 7.2. Understandability

This proposal decouples NFC hardware elements from its representation in code.

The use of the proposed MDA improves understandability because it enables developers to focus on the NFC elements of the application by means of a model. The metamodel supports the definition of different models to represent the application behaviour. It allows designers to distribute the behaviour of the system easily.

In fact, due to the definition of a DSL to represent NFC-based applications in terms of high level concepts, such as interaction surface, hot spot, tag, and so on, developers do not deal with low level concepts such as the NFC reader, HTTP requests and responses, etc. providing them with a high-level view of the system that can be modified easily.

### 7.3. Accuracy

As we mentioned in Section 7.1, the definition of NFC-based application models hides NFC hardware complexity from developers. The example shown in Section 6 describes how developers use the defined DSL to define a model, and how the proposed model is transformed into text (source code).

### 7.4. Predictiveness

The definition of the proposed metamodel improves the prediction of specific system behavior introducing validation at the metamodel level—for instance, to check that tags mappings are not missing.

Thus, the validation is defined once for all models, improving model reliability, reuse and reducing maintenance costs; for instance, new invariants can be easily introduced without modifying the transformation function definition.

It improves model predictiveness because model validation ensures that the generated source code is valid.

### 7.5. Inexpensiveness

The definition of the metamodel enables developers to generate source code automatically. This technology makes the generation of complex applications less expensive.

### 7.6. Benefits and Weaknesses

The previous sections have shown that the proposed process accomplishes the five dimensions of model-driven architecture.

Thus, the main benefit of the proposal is that it allows developers to create mixed environments where the availability and flexibility of NFC based interaction systems can be easily configured, extended and maintained.

The main weakness is the complexity of the proposed tools. This process requires developers to have special skills in MDA, OCL validation and transformation languages. Developers have to deal with abstraction, modeling and non-trivial tools.

## 8. Conclusions 

The main contribution of this paper is the definition of a Model Driven Architecture (MDA) to model different configurations of NFC-based applications. The MDA defines a Platform Independent Model (PIM) and a Platform Specific Model (PSM). A complete MDA definition should include the possibility to transform models into code. This proposal also includes a model to text transformation.

Designers use the PIM to model the application physical surfaces and hot spots jointly with the application model and actions that are performed on it.

The PSM defined by the MDA allows designers to configure how the physical surfaces are identified and the location where model actions are executed. This model allows developers to create mixed environments where the availability and flexibility of NFC based interaction systems can be easily configured.

The model to text transformation allows the generation of different applications according to the PSM configuration. These applications belong to three different types:

- The User Application that allows final users to interact with the system,
- The Server Application that allows the definition of distributed NFC based applications,
- The Configuration Application that eases the deployment process of this NFC-based applications.

The whole process is supported by Eclipse Modeling Tools, which provides developers and designers with a robust development environment. In addition, the process follows the OMG standards to encourage the interoperability of the system and third party tools. The model to text transformation, which is written in Acceleo, proved to generate most of the common code for different applications.

The main benefit of the proposal is that it allows developers to create mixed environments where the availability and flexibility of NFC-based interaction systems can be easily configured, extended and maintained.

Traditional approaches force developers to build applications for different platforms (e.g., Web, iOS, Android, Windows, etc.), leading to different development branches that are prone to errors, and difficult to maintain and test (e.g., changes and fixes on the application domain model should be addressed in all platforms).

Model-driven architectures decouple application functionality from technology, which enables developers to create Platform Independent Models (PIMs) and Platform Specific Models (PSMs) to derive application source code semi-automatically using model transformations.

The use of PIMs enables developers to:

- Change the application model once and propagate these changes to the PSMs automatically using model-to-model transformations. This process reduces the maintenance costs and minimizes the divergence in the development of the application for different platforms.
- Keep the project documentation synchronized with the source code of the application, since it is derived from models using model to text transformations.
- Verify model integrity, which leads to the semi-formal validation of the application at the domain level using OCL constraints.

One of the main features of employing MDAs is the design time interoperability. This feature captures different application concerns in independent models, which are integrated at the last stage of development (just before the generation of the application source code).

As future work, two research lines are planned: measuring the amount of code generated according to the domain of the application that is generated; and the integration of this framework and MDA with other UI modelling languages to improve the amount of code to be generated by the architecture.

## References

[1] ISO Information Technology—Automatic Identification and Data Capture Techniques—R Code Bar Code Symbology Specification. https://www.iso.org/standard/62021.html.

[2] Tesoriero R., Gallud J.A., Lozano M.D., Penichet V.M.R.P. (2014). Enhancing visitors’ experience in art museums using mobile technologies. Inf. Syst. Front..

[3] Tesoriero R., Villanueva P.G., Fardoun H.M., Sebastián G. (2014). Distributed user interfaces in public spaces using RFID-based panels. Int. J. Hum. Comput. Stud..

[4] Tesoriero R., Fardoun H.M., Gallud J.A., Lozano M.D., Penichet V.M.R.P. Interactive Learning Panels. Proceedings of the of 13th International Conference Human-Computer Interaction (HCI), Interacting in Various Application Domains.

[5] Kleppe A., Warmer J., Bast W. (2003). MDA Explained: The Model Driven Architecture.

[6] (2018). Object Management Group. http://www.omg.org/.

[7] Fielding R. (2000). Chapter 5: Representational State Transfer (REST). Architectural Styles and the Design of Network-Based Software Architectures.

[8] Mellor S.J. (2004). MDA Distilled, Principles of Model Driven Architecture.

[9] Object Management Group (OMG) OMG Unified Modeling Language™ (OMG UML) Version 2.5. https://www.omg.org/spec/UML/2.5.1/PDF.

[10] Object Management Group (OMG) OMG Meta Object Facility (MOF) Core Specification Version 2.5.1. http://www.omg.org/spec/MOF/2.5.1/PDF.

[11] Object Management Group (OMG) XML Metadata Interchange (XMI) Specification Version 2.5.1. http://www.omg.org/spec/XMI/2.5.1/PDF.

[12] Object Management Group (OMG) Common Warehouse Metamodel (CWM) Specification. https://www.omg.org/spec/CWM/1.1/PDF.

[13] Puerta A. (1997). A model-based interface development environment. IEEE Softw..

[14] Feuerstack S., Pizzolato E.B. (2012). Engineering Device-Spanning, Multimodal Web Applications Using a Model-Based Design Approach.

[15] Manca M., Paternò F., Santoro C., Spano L.D. (2013). Generation of Multi-Device Adaptive MultiModal Web Applications. Proceedings of the 10th International Conference on Mobile Web and Information Systems, MobiWIS 2013.

[16] Paternò F., Santoro C., Spano L.D. (2011). Engineering the authoring of usable service front ends. J. Syst. Softw..

[17] Blumschein P., Hung W., Jonassen D., Strobel J. (2009). Model-Based Approaches to Learning: Using Systems Models and Simulations to Improve Understanding and Problem Solving in Complex Domains.

[18] Koch N., Kraus A. (2003). Towards a Common Metamodel for the Development of Web Applications.

[19] Retalis S., Papasalouros A., Skordalakis M. (2002). Towards a Generic Conceptual Design Metamodel for Web-Based Educational Applications. Proceedings of the IWWWOST’02.

[20] ISO (2011). Identification Cards—Contactless Integrated Circuit Cards—Proximity Cards—Part 3: Initialization and Anti-Collision.

[21] ISO (2009). Identification Cards—Contactless Integrated Circuit Cards—Vicinity Cards—Part 3: Anti-Collision and Transmission Protocol.

[22] Gamma E., Helm R., Johnson R., Vlissides J. (1995). Design Patterns: Elements of Reusable Object-Oriented Software.

[23] Eclipse Foundation (2016). Eclipse Modeling Framework. http://www.eclipse.org/modeling/emf/.

[24] Eclipse Foundation (2016). Eclipse Modeling Project. https://eclipse.org/modeling/.

[25] Object Management Group (2006). Object Constraint Language Specification Version 2.0. http://doc.omg.org/formal/06-05-01.pdf.

[26] Eclipse Foundation (2016). Acceleo. https://eclipse.org/acceleo/.

[27] Selic B. (2003). The pragmatics of model-driven development. IEEE Softw..

